# Genome Sequence of the Alkaliphilic Bacterial Strain *Bacillus ligninesis* L1, a Novel Degrader of Lignin

**DOI:** 10.1128/genomeA.00042-13

**Published:** 2013-03-07

**Authors:** Daochen Zhu, Pingping Li, Shoko-Hosoi Tanabe, Jianzhong Sun

**Affiliations:** a Biofuels Institute, School of Environmental Engineering, Jiangsu University, Zhenjiang, China; b Institute of Agricultural Engineering, Jiangsu University, Zhenjiang, China; c Department of Ecosystem Studies, School of Environmental Science, University of Shiga Prefecture, Hikone, Shiga, Japan

## Abstract

*Bacillus ligninesis* strain L1, isolated from seafloor sediment, was able to grow on medium with lignin as its sole carbon source. Here, we report a 3.8-Mbp high-quality genome sequence for this bacterium. The genes involving ectoine and glycine betaine synthesis, as well as those involved in the degradation of lignin, were identified.

## GENOME ANNOUNCEMENT

An alkaliphilic and halotolerant Gram-positive bacterium strain, *Bacillus ligninesis* L1, was isolated from sediment samples at a benthal depth of 3,000 m in the South China Sea. The strain L1 grew and adapted well at a wide range of temperatures (10.0 to 50.0°C) and pH levels (6 to 11.5), with the optimal growing conditions being 30°C and pH 9.0. Strain L1 is able to utilize lignin as its sole carbon source, which presents its potential value in the cellulosic biofuels industry.

The whole genome sequence of *B. ligninesis* L1 was obtained using the Illumina/Solexa HiSeq 2000 sequencing system at Majorbio BioTech (China) with a paired-end library. A total of ~833 Mb of paired reads with about 213-fold coverage of the genome was generated, which was then trimmed to ~439 Mb of paired reads and assembled into 241 contigs and 72 scaffolds using the Short Oligonucleotide Alignment Program (SOAP)*denovo* software (1). The open reading frames (ORFs) were identified with the GeneMark gene prediction tool (http://exon.gatech.edu/GeneMark) and further analyzed using BLAST to screen the nonredundant protein database, and the relevant gene functions were determined with the help of the Clusters of Orthologous Genes (COGs) (2) and Kyoto Encyclopedia of Genes and Genomes (KEGG) databases. Following these data analyses, the genes encoding tRNA and rRNA were eventually obtained using the RNAmmer software.

The genome of strain L1 is 3,862,148 bp long with a G+C content of 40.76% and contains 4,101 open reading frames, of which 2,847 ORFs have positive biological functions. In addition, a total of 4,101 coding sequences (CDSs) were identified (of which 2,505 CDSs were for putative biological functions), including 72 tRNA genes and 7 rRNA operons in the genome.

The strain L1 genome contains four gene clusters involved in compatible solute synthesis, including two ectoine synthesis gene clusters and two glycine betaine synthesis genes, which potentially enhance cell survival in high salinity or other extreme environments (3, 4). The relevant genes associated with lignin degradation were also identified in the strain L1 genome, including two aryl-alcohol dehydrogenase genes, 19 NADH dehydrogenase genes (5), two glycolate oxidase genes (6), and one Mn-superoxide dismutase gene (7).

This genome information suggests that L1 has a function in degrading lignin substrates that might offer a unique value for the cellulosic biofuels industry.

### Nucleotide sequence accession number.

This Whole Genome Shotgun project has been deposited at DDBJ/EMBL/GenBank under the accession no. ANNK00000000.
